# Perceived social support and associations with health-related quality of life in young versus older adult patients with haematological malignancies

**DOI:** 10.1186/s12955-019-1202-1

**Published:** 2019-08-22

**Authors:** Kristina Geue, Heide Götze, Michael Friedrich, Katja Leuteritz, Anja Mehnert-Theuerkauf, Annekathrin Sender, Yve Stöbel-Richter, Norbert Köhler

**Affiliations:** 1 Department of Medical Psychology and Medical Sociology, University of Leipzig, Philipp-Rosenthal-Str. 55, 04103 Leipzig, Germany; 2 University of Zittau / Goerlitz, Faculty of Managerial and Cultural Studies, 30 06 48 Goerlitz, Germany; 3 University of Leipzig, Clinical Trial Centre Leipzig, Coordinating Centre for Clinical Trials, Härtelstraße 16/18, 04107 Leipzig, Germany

**Keywords:** AYA, Older adult patients, Quality of life, EORTC, Oncology

## Abstract

**Background:**

This study compared the perceived social support of young and older adult cancer patients, examining possible influencing factors as well as associations with health-related quality of life.

**Methods:**

A total of 179 young patients (18–39 years) and 200 older adult patients (> 70 years) with haematological malignancies completed questionnaires on their perceived social support (ISSS-8, scales: Positive Support and Detrimental Interactions, range 0–16) and health-related quality of life (EORTC QLQ-C30). Tests for mean differences, correlations and regression analyses to determine associated variables of social support were performed.

**Results:**

No difference was reported between young (M = 13.40, SD = 2.81) and older adult patients (M = 13.04, SD = 3.82; *p* = .313) for Positive Support. However, young patients (M = 4.16, SD = 3.10) reported having had more Detrimental Interactions than older patients did (M = 1.63, SD = 2.42; *p* < .001, Cohen’s d = .910). Comparison of the EORTC QLQ-C30 Function scales showed poorer outcomes for young patients on Emotional, Cognitive and Social Functions and a higher outcome on Physical Function compared with older adult patients. Regression analyses indicated that age (young vs. older adult patients) significantly explained proportions of variance in all models, with young age having a negative impact on Emotional, Cognitive and Social Functions and a positive impact on Physical and Role Functions compared with old age. Significant associations between Detrimental Interactions and all the scales examined except Cognitive Function were found.

**Conclusions:**

The difference in negative perceptions of social support in young vs. older adult patients and its impact on health-related quality of life emphasises the necessity of differentiating between positive and negative social support. Negative interactions should be addressed through psychosocial care, particularly with young cancer patients.

## Background

Haematological malignancies comprise three major diagnosis groups: leukaemia (acute myelogenous or acute lymphoblastic), lymphoma (Hodgkin’s lymphoma and non-Hodgkin’s lymphoma) and plasma cell neoplasms (multiple myeloma). Each year, about 230,000 people in Europe are diagnosed with haematological malignancies, and the overall incidence appears to be on the rise [1]. Haematologic malignancies are most common among older adults, the median age for such conditions being 69 years [1, 2]. However, Hodgkin’s lymphoma and leukaemia are among the five major cancer diagnoses common in adults under 40 [3]. The National Cancer Institute defined individuals aged 15 through 39 years at cancer diagnosis as adolescent and young adult patients (AYA) [4]. Epidemiological and therapeutic outcomes are not the only things that significantly set AYA patients apart from older, haematological cancer patients [5]. Each developmental stage across the life span has its own developmental tasks and related challenges. Younger people also face challenges that are specific to their stage of life and which consequently differ from those dealt with by older people.

Young adulthood is a phase of life marked by change and upheaval [6]. Some of the developmental tasks associated with this period include, for example, forming financial and social independence, detaching from parents, the formation and consolidation of romantic relationships, family planning and family formation, as well as establishing a professional career [7, 8]. Creating and firmly establishing their own social network is another important development young people undergo. In contrast, late adulthood is characterised by learning how to cope with increased frailty, physical comorbidities, functional restrictions, cognitive deficits and the inability to perform everyday activities [9–11].

With regard to health-related quality of life in the general population, the following can be noted: Older adults (> 70 years) have a lower level in physical, role, social and cognitive functioning and more physical symptoms such as fatigue and insomnia compared to young adults [12]. Hall et al. [13] showed that young cancer patients aged between 18 and 40 years had significantly lower levels of social functioning, but higher levels of physical functioning than those reported by their older counterparts (> 64 years). In Drost et al.’s study [14], young adult lymphoma survivors reported having higher health awareness and positive self-evaluation than did older adult patients (> 65 years), while no significant differences were observed regarding appearance and body change concerns or worries. Furthermore, older cancer patients were less interested in offers of psychological support [15] and had lower supportive care needs than did young patients [13].

Social support is important for cancer patients, and many psycho-oncological studies have shown that social support improves their ability to cope with the disease, decreases disease-related stress, increases well-being and health-related quality of life, and encourages self-esteem [16–20]. Despite this, only a few studies conducted so far have examined social support in young cancer patients. Social support is defined as perceived or objectively existing resources available to a person in his or her social network. A distinction can be made between positive and negative social support. However, little research has been conducted on negative aspects of social interactions [21]. Social networks change over a person’s lifespan: on the one hand, ageing generally goes hand in hand with one’s social circles getting smaller; on the other, the number of emotionally relevant people in a person’s life tends to remain rather stable [7]. Adult cancer patients (all age groups, average age: 62 years) usually consider their partner or spouse to be their most important source of social support [22, 23]. For younger cancer patients, two studies identified both friends and family as effective sources of social support [24, 25]. Little is known currently, however, about perceived social support, particularly negative social support, stemming from young and older adult cancer patients’ social networks, and its associations with health-related quality of life. Learning more about this is a necessary component of providing adequate professional, psychosocial, support to patients and their most important caregivers - those who are uniquely well positioned to offer positive social support and reduce negative support patterns.

Therefore, we addressed the following research questions:

(1) What are the levels of self-perceived social support (positive and negative) in young vs. older haematological cancer patients? What differences exist between those two groups?

(2) What influence do sociodemographic variables have on self-perceived social support in haematological cancer patients?

(3) Which dimensions of health-related quality of life are different in young vs. older haematological cancer patients? What association exists between self-perceived social support and health-related quality of life?

## Methods

### Design

#### Young adult patients (AYA)

Data were collected as part of a prospective, longitudinal study. In this study, young adult cancer patients were surveyed at two measuring time points using the baseline survey (t1). Participants had to meet the following inclusion criteria: (1) age at diagnosis: 18 to 39 years (age range based on the widest age range by the National Cancer Institute [4] minimum age of 18 years was chosen due to the legal requirement in Germany for patients to be 18 years old to participate in studies without the written consent of their parents); (2) first manifestation of cancer (all malignant tumour identities); (3) diagnosis made within the previous four years. The inclusion criterion for the haematological sample was: first manifestation of haematological neoplasm (ICD-10: C81–C96).

The recruitment of participants was carried out from May 2014 to December 2015 (t1) throughout Germany, in co-operation with 16 oncological, acute care hospitals, four (cancer) rehabilitation centres and two cancer registries. Potential candidates were informed about the research project via the following media forms: flyers, posters and the project’s homepage and Facebook page. Once a candidate agreed to participate, they were either given a link to the standardised study questionnaire or sent a hard copy questionnaire by mail. Participants received an allowance of 10€ for completing the questionnaire. More information about the study has been provided by Leuteritz et al. [26].

#### Older adult patients

The older adult patients’ data was sourced from the baseline survey of a prospective, longitudinal, epidemiological study with three measuring points. Patients who participated in that study had to meet the following criteria: (1) age > =70 years (due to the lack of a fixed age limit for older adult patients, the age limit based on gerontological age concepts [27], the time of transition to retirement), (2) manifestation of haematological cancer (ICD-10: C81–C96), (3) and diagnosis made within the previous five years.

Patients eligible for enrolment were treated in three Leipzig-based hospitals: one university hospital, and two general hospitals. Baseline recruitment began in August 2014 and was completed in May 2016. Patients eligible for study participation received an invitation letter with information about the study. Those who agreed to participate received a phone call in order to make an appointment for interview. Patients who did not wish to be personally interviewed were sent a postal questionnaire. Study participants were offered an allowance of 10€. More information about the 70+ study has been provided by Köhler et al. [28].

Both studies received research ethics committee approval (Ref.-Nr. 372–13-16122013 and 071–14-10032014) from the University of Leipzig medical faculty ethics board.

### Measures

Self-report questionnaires were used to assess socio-demographic and disease-related medical data. The socio-demographic variables collected were: age, gender, partnership, children and education. Medical data included cancer diagnosis, time of diagnosis and medical treatments.

#### Illness-specific social support scale short version-8 (ISSS-8)

The German version of the Illness-specific Social Support Scale was adapted by Ramm and Hasenbring [29] from the original version by Revenson et al. [30]. The eight-item German version of the Illness-specific Social Support Scale (ISSS) aims to assess self-perceived positive and negative social support in patients with chronic diseases. The complete instruction for the ISSS-8 is: “These questions are about your relationships with important people: your partner, family members, friends and acquaintances, colleagues and neighbours. We want to understand how you experience and evaluate these relationships. Sometimes the behaviour of others is very helpful for us, sometimes less, sometimes stressful. Below are some of the behaviors that people can show when someone is sick. Please tick how often one or more of your related persons have behaved towards you in this way.”

The ISSS-8 [31] includes the two scales ‘Positive Support’ (four items) and ‘Detrimental Interactions’ (four items) for negative social support. Items are scored on a five-point Likert scale ranging from 0 (‘never’) to 4 (‘always’), with a score range of 0 to 16. While on the ‘Positive Support’ subscale higher scores indicate better support, higher scores on the ‘Detrimental Interactions’ subscale reflect more pronounced negative interactions between the patient and his or her loved ones [31]. According to Revenson et al. [30], positive and negative interactions are unrelated. The two scales show internal consistencies with Cronbach’s alpha = .88 and .68 respectively [29].

#### European organisation for the research and treatment of Cancer quality of life questionnaire-Core 30 (EORTC QLQ-C30)

The EORTC QLQ-C30 measures health-related quality of life (HRQoL) and comprises five multi-item functioning scales - Physical, Role, Emotional, Cognitive and Social; three multi-item symptom scales (Fatigue, Nausea, Pain); six single-item symptom scales (Dyspnoea, Insomnia, Appetite loss, Constipation, Diarrhoea and Financial Difficulties); and, one multi-item global quality of life scale. Scores range from 0 to 100. For the functional scales, higher values indicate better functioning; for the symptom scales, higher values represent a higher burden of symptoms [32].

The clinical significance of point differences in the scores can be determined by differentiating between trivial, small, medium and large effects. The cut-off-points for clinical interpretation are presented for each individual scale in the guidelines for individual scales set by Cocks et al. [33].

### Data analysis

All analyses were performed with the software package R (version 3.4.2). Descriptive statistics were calculated (mean and standard deviation) to describe levels of social support and health-related quality of life. We assessed significance levels of differences between means using the t-test for independent samples. To account for multiple testing, the *p*-value was adjusted to *p* = 0.0029412 (Bonferroni). Multiple linear regression models were computed to estimate the impact of relevant sociodemographic factors (AYA vs. older adult patients, gender, partnership (yes vs. no) and parental status (yes vs. no) on both ISSS-8 scores (Positive Support and Detrimental Interactions). Furthermore, the impact of group (AYA vs. older adult patients), gender, partnership (yes vs. no), Positive Support and Detrimental Interactions on each of the EORTC functional scores and the Global quality of life score were estimated using multiple linear regression models. Correlations between ISSS-8 and EORTC scales were computed using the Pearson correlation coefficient.

## Results

### Sample

A total of 179 AYA patients and 200 older adult patients participated in the study (Table 1). The mean age was 27.5 years (SD = 5.8) for the AYA sample and 76.1 years (SD = 4.9) for the older adult patients. The majority of older adult patients, in contrast to the young patients, were male (64.0% vs. 38.5%; *p* < .001). older adult patients were more likely than the young adults (*p* < .001) to have a partner and children. The most frequent diagnosis in both groups was lymphoma (AYA: 76.5%; older adults: 51.5%) and the majority of patients were treated with chemotherapy (AYA: 97.1%; older adults:: 77.0%).

**Table 1:** Sociodemographic and clinical characteristics of the samples

	Young adult patients *N* = 179 N (%)	Older adult patients *N* = 200 *N* (%)
*Sociodemographic*		
Age* (mean, standard deviation)	27.5 (5.79)	76.1 (4.91)
range in years	19–42	70–96
Gender*		
male	69 (38.5)	128 (64.0)
female	120 (61.5)	72 (36.0)
Family status*		
single	141 (78.8)	7 (3.5)
married	35 (19.6)	144 (72.0)
divorced	3 (1.7)	12 (6.0)
widowed	–	37 (18.5)
Partnership* yes	78 (43.6)	150 (75.0)
Children* yes	32 (17.9)	184 (92.0)
Education*		
none	4 (2.2)	1 (0.5)
8 years	14 (7.9)	84 (42.0)
10 years	56 (31.5)	43 (21.5)
A-level	22 (12.7)	4 (2.0)
University	82 (46.1)	68 (34.0)
*Clinical characteristics*		
Cancer diagnosis*		
Lymphoma	137 (76.5)	103 (51.5)
Leukaemia	38 (21.2)	54 (27.0) 43 (21.5)
Myeloma	4 (2.2)	
Medical therapies *(Multiple answers possible)*		
Chemotherapy*	175 (97.8)	154 (77.0)
Radiotherapy	79 (44.1)	60 (30.0)
Transplantation (stem cell and/or bone marrow)	27 (15.1)	33 (16.5)

*significant difference between AYA and older patients with *p* < .001

### Social support (ISSS-8)

The mean value of the Positive Support score was 13.21 (SD = 3.4). No significant difference was observed between the two groups (AYA: M = 13.4, SD = 2.8; older adult patients: M = 13.0, SD = 3.8; *p* = .313) (Table 2). The maximum value of 16 was reached by 80 older adult patients (40.0%) vs. 52 AYA patients (29.1%).

**Table 2:** Mean and standard deviation for social support (scale and items)

ISSS-8	Young adult patients (*N* = 179)		Older adult patients (*N* = 200)	
Amongst the people you feel close to, is there someone who...	Mean	SD	Mean	SD
Positive Support	**13.40**	**2.81**	**13.04**	**3.82**
Is there for you when you need him/her	3.8	0.6	3.8	0.6
Gives you comfort	3.4	0.9	2.9	1.5
Talks about important decisions with you	3.1	1.1	3.3	1.3
Spends part of his/her time working some things out for you	3.2	1.1	3.1	1.4
Detrimental Interactions	**4.16**	**3.10**	**1.63**	**2.42**
Worries too much about your illness	1.4	1.1	0.8	1.2
Gives you information or makes suggestions that you find unhelpful or upsetting	1.3	1.1	0.4	0.9
Makes you feel you cannot care for yourself	0.8	1.2	0.3	0.8
Tries to change the way you’re coping with your illness in a way you don’t like	0.6	0.9	0.1	0.4

*SD* Standard deviation

Bold numbers show scale values

The mean value of the Detrimental Interactions score was 2.8 (SD = 3.0). AYA patients reported stronger Detrimental Interactions than older adult patients did (AYA: M = 4.2, SD = 3.1; older adult patients: M = 1.6, SD = 2.4; *p* < .001, Cohen’s d of 0.9). A total of 54.0% (*N* = 108) of the older adult patients reported having had no Detrimental Interactions (score = 0) compared with 12.3% (*N* = 22) of the AYA patients. Regression analyses showed that partnership (β = 2.3; *p* < .001) had a significant effect on Positive Support, while the other factors included had no significant effect (age group, AYA vs. older adult patients, gender and having children (*R*^2^ = 0.10)) (Table 3). We found a significant association between the factors Detrimental Interactions and age group (β = 2.6; *p* < .001), whereby young patients had higher scores than those of older adult patients. None of the other sociodemographic factors included were significantly associated with the Detrimental Interactions score. The whole model explained about 18% of variance (adjusted *R*^2^ = 0.18).

**Table 3:** Mean and standard deviation for health-related quality of life

*HRQoL (EORTC QLQ-C30)*	Young adult patients (*N* = 179)		Older adult patients (*N* = 200)		Group differences	Effect size	mean differences – clinical relevance*	
	Mean	SD	Mean	SD	*p*	d		
*Function scales*								
Physical Function	79.59	(18.06)	67.73	(27.79)	*<.001*	.516	11.86	small
Role Function	64.25	(26.33)	60.75	(36.64)	.292	.11	3.50	trivial
Emotional Function	59.50	(27.71)	78.04	(20.70)	*<.001*	.76	−18.54	–
Cognitive Function	69.46	(25.08)	80.25	(22.48)	*<.001*	.45	−10.79	medium
Social Function	57.45	(32.77)	74.75	(31.88)	*<.001*	.54	−17.30	large
*Symptom scales*								
Fatigue	46.99	(25.06)	43.72	(31.21)	.265	.12	3.27	trivial
Nausea/vomiting	7.08	(16.27)	7.25	(17.17)	.920	.00	−0.17	trivial
Pain	22.63	(27.46)	34.00	(33.91)	*<.001*	.34	−11.37	small
*Single items*								
Dyspnoea	24.58	(29.63)	25.67	(33.70)	.741	.03	−1.09	trivial
Insomnia	35.01	(34.49)	34.00	(38.39)	.789	.03	1.01	trivial
Appetite loss	13.22	(23.55)	21.17	(33.61)	.009	.27	−7.95	small
Constipation	5.40	(15.86)	15.00	(28.11)	*<.001*	.42	−9.60	small
Diarrhoea	14.15	(25.20)	9.33	(22.46)	.050	.20	4.82	small
Financial difficulties	44.13	(37.33)	7.50	(20.17)	*<.001*	1.22	36.63	medium
*Global quality of life*	68.39	(17.84)	58.50	(22.28)	*<.001*	.49	9.89	small

*SD* Standard deviation, *EORTC QLQ-C30* European Organisation for the Research and Treatment of Cancer Quality of Life Questionnaire -

Core 30, * = by Cocks et al. (2011)

### Health-related quality of life (EORTC QLQ-C30)

Regarding Global quality of life, AYA patients had higher scores (68.4) than those of older adult patients (58.5) (Table 4). However, for the functional scales the former had worse Emotional, Cognitive and Social Function scores but higher Physical Function scores. The groups’ symptom scores also differed on the Pain subscale, whereby older adult patients scored higher than AYA patients. Furthermore, young adult cancer patients reported having more Financial Difficulties than older adult patients did, with the scores on this scale showing the greatest difference between the two groups (mean difference 36.6 with Cohen’s d = 1.2).

**Table 4:** Regression analyses for Social Support (ISSS-8)

	beta	std. error	*p*-value
*Positive Support (adj. R*^*2*^ *= 0.10)*			
(Intercept)	12.05	0.57	< .001
Age group: AYA	0.52	0.51	.30
Gender: male	−0.16	0.35	.64
Partnership: yes	*2.31*	*0.37*	*< .001*
Children: yes	−0.69	0.52	.19
*Detrimental Interactions (adj. R*^*2*^ *= 0.18)*			
(Intercept)	1.47	0.49	.003
Age group: AYA	*2.62*	*0.43*	<.001
Gender: male	0.24	0.30	.41
Partnership: yes	−0.08	0.32	.79
Children: yes	0.07	0.45	.87

### Association between social support and health-related quality of life

#### Positive support

Correlations between Positive Support and HRQoL (all scores and single items) higher than *r* = .10 for the total group were observed for Physical Function (*r* = .11; *p* < .05) and Role Function (*r* = .10; *p* < .05) (Table 5). The highest correlations seen for older adult patients were *r* = − 0.19 for Physical Function (*p* < .01), Social Function (*p* < .01) and Positive Support. For the young adult patients, Emotional Function and Positive Support had the highest correlation, with *r* = .16 (*p* < .05).

**Table 5:** Correlation matrix for Social Support and health-related Quality of Life

	Positive Support			Detrimental Interactions		
	Total group	AYA patients	Older patients	Total group	AYA patients	Older patients
*HRQoL (EORTC QLQ-C30) Function scales*						
Physical Function	−.108*	.032	−.192**	−.025	−.010	−.191**
Role Function	−.102*	.053	−.181*	−.161**	−.249***	−.180*
Emotional Function	.067	.164*	.035	−.2960***	−.173*	−.173*
Cognitive Function	.060	.120	.041	−.149**	−.165*	.065
Social Function	−.083	.102	−.192**	−.370***	−.315***	−.282***

* *p* < .05, ** *p* < .01, ***p < .001

#### Detrimental interactions

For the total group, correlations higher than *r* = 0.20 were found for Detrimental Interactions and Emotional Function (*r* = .30; *p* < .001), Social Function (*r* = .37; *p* < .001) and Financial Difficulties (*r* = .35, *p* < .001). For both groups individually, the highest correlation observed was that between Social Function and Detrimental Interactions (AYA: *r* = −.32; *p* < .001; older adult patients: *r* = −.28; *p* < .001).

Regression analyses were conducted for Global quality of life and the five Function Scales (Table 6). We found significant associations between Detrimental Interactions and all the scales examined except for Cognitive Function.

**Table 6:** Regression analyses for health-related quality of Life

	beta	std. error	p-value
*Global Quality of Life (adj. R*^*2*^ *= 0.09)*			
(Intercept)	61.95	4.43	*< .001*
Age group: AYA Partnership: yes	13.32–2.03	2.42 2.34	*< .001*.39
Gender: male	0.48	2.15	.82
Positive Support	0.02	0.32	.94
Detrimental Interactions	−1.56	0.37	*< .001*
*Physical Function (adj. R*^*2*^ *= 0.09)*			
(Intercept)	78.01	5.17	*< .001*
Age group: AYA Partnership: yes	16.24–1.45	2.83 2.73	*< .001*.60
Gender: male	5.44	2.50	*.03*
Positive Support	−0.82	0.37	*.03*
Detrimental Interactions	−1.25	0.43	*.004*
*Emotional Function (adj. R*^*2*^ *= 0.18)*			
(Intercept)	67.64	5.23	*< .001*
Age group: AYA Partnership: yes	−13.96-4.28	2.86 2.76	*< .001*.12
Gender: male	8.55	2.53	*< .001*
Positive Support	0.82	0.38	.03
Detrimental Interactions	−1.60	0.44	*< .001*
*Cognitive Function (adj. R*^*2*^ *= 0.05)*			
(Intercept)	71.88	5.25	*< .001*
Age group: AYA Partnership: yes	−8.72-1.45	2.88 2.78	*.003.60*
Gender: male	4.80	2.55	.06
Positive Support	0.56	0.38	.14
Detrimental Interactions	−0.59	0.44	.18
*Social Function (adj. R*^*2*^ *= 0.15)*			
(Intercept)	88.42	6.83	*< .001*
Age group: AYA Partnership: yes	−9.74-7.39	3.74 3.61	*.01.04*
Gender: male	3.48	3.31	.29
Positive Support	−0.36	0.49	.47
Detrimental Interactions	−3.51	0.57	*< .001*
*Role Function (adj. R*^*2*^ *= 0.04)*			
(Intercept)	76.59	6.99	*< .001*
Age group: AYA Partnership: yes	9.30–3.43	3.83 3.69	*.02*.35
Gender: male	2.41	3.39	.48
Positive Support	−0.84	0.50	.10
Detrimental Interactions	−2.36	0.59	*< .001*

Furthermore, age significantly explained proportions of variance in all the regression models, whereby younger age had a negative impact on Emotional, Social and Cognitive Function, and a positive impact on Physical and Role Function. The analyses also showed female patients to have a lower Physical and Emotional Function than those of male patients. A relationship was observed between Positive Support and Physical Function, as well as partnership and Social Function. The regression models for Emotional and Social Function had the highest explanatory power, at 18 and 15% respectively.

## Discussion

This study compares levels of perceived social support among young and older adult haematological cancer patients and provides initial indications of differences between the groups. It also identifies associations between social support and health-related quality of life.

### Perceived social support

The study participants, both AYA and older adult patients, reported high levels of perceived positive social support. In line with these results, Hann et al. [34] reported no correlation between age and social support in cancer patients diagnosed with a variety of tumour entities (see also Pinquart et al. [35] for patients with acute myeloid leukaemia (AML) and Soares et al. [36] for Hodgkin lymphoma survivors). Living in partnership was a predictor of high levels of positive social support in our sample (AYA and older patients). Pailler et al. [37] also showed a positive correlation between social support and marital satisfaction among AML patients.

Regarding perceived negative social support, we found higher levels in AYA patients than in older patients. No further sociodemographic predictors for negative social support were found. For the Danish population, Due et al. [38] reported significant age differences in the structure and function of social relations. In conclusion, older patients seem to develop a restricted social network over their lifespan close only to those people who provide purely positive support [38]. Furthermore, according to Erickson’s development theory, younger and middle-aged people are in an active phase of life that is oriented towards the outer world and the future. By contrast, older people tend to be more inwardly oriented and focus on the ‘here and now’ [39]. Young people have more social contacts from different areas of life than older people [38]. Thus, the chance for detrimental interactions is higher for younger than for older patients. Finally, the presence of negative social support among the young might also be related to the fact that developing adequate and sufficient coping strategies is a process that can take a lifetime. Older adult patients seem to become more pragmatic and reluctant to deal with interpersonal conflicts [38].

### Health related quality of life

It must be stated that age group (AYA vs. older adult patients) had a significant impact on all HRQoL function scales: Compared with older adult patients, AYA patients had lower levels of Emotional, Cognitive and Social functioning. Only for the Physical function scale and global quality of life scale, we found higher levels for the AYA patients compared to older patients. In the general population health related quality of life, significantly decreases in all domains with age (exception: emotional function) [12]. Furthermore, the higher level of financial burden experienced by the AYA patient group indicates that cancer has a substantial financial impact on young adults [13]. Guy et al. [40] pointed out that young adults with cancer have significantly higher health expenditure and productivity losses compared to adults without cancer in prehistory or to older adult cancer survivors, even a long time after the diagnosis of cancer. At diagnosis, AYA patients were often completing higher education, entering a new job and/or raising young children, had less financial reserves, and, thus, have a higher socioeconomic burden [41].

### Associations between social support and health-related quality of life

Previous studies have shown a positive association between higher perceived social support and HRQoL for various tumour entities [35–37, 42]. Hann et al. [34] reported larger social support networks to be associated with less depression in younger patients (18–54 years), but not in older adult patients (> 54 years). Our results showed stronger correlations between negative social support and HRQoL than between positive social support and HRQoL. Thus, detrimental interactions generally had a negative impact on HRQoL. In contrast, positive social support was found to have an impact only on Emotional and Physical functioning. Our findings support the moderator model hypothesizing that positive social interactions buffer the negative effects of detrimental interactions on quality of life [21]. While positive support is considered self-evident, detrimental interactions are experienced as more significant events than positive interactions. However, negative social support seems to be an important indicator of HRQoL.

The existence of a social network does not necessarily indicate more positive support. This should be considered when providing medical and psychosocial care to cancer patients and their caregivers. In this context, it is interesting to note that living in a partnership is negatively associated with Social Function. This finding may be rooted in methodological issues, because the two items on the scale Social Functioning refer to family life or to social activities with other peoples as a whole, while partnership is not named. However, it may also be an indication that positive and negative support can simultaneously come from the same source. Furthermore, it would be interesting to learn whether there are relevant associations between coping strategies, mental health and perceived social support.

### Limitations

By including patients with different rates of hematological malignancies in the two samples, the sample homogeneity of this study was limited. There were also differences in sociodemographic characteristics (e.g. partnership, children, and gender). One reason for the different gender proportions in the two groups is the higher incidence of haematological malignancies in the female vs. male AYA patients. In contrast, more men than women suffer from haematological malignancies in late adulthood. Although we tried to control for these differences in the regression models, it remains unclear whether age and/or being in a partnership are relevant social support factors.

The chosen approach of the chronological age categories for group classification may give the impression of two homogeneous groups. However, the tasks of the described developmental phases for young vs. late adulthood (e.g. raising a family, dealing with physical restrictions) is, however, very different from individual to individual and depends on many factors. This means that there is heterogeneity within both of the two age groups. However, a classification according to developmental task does not seem to be methodically feasible.

The ISSS-8 questionnaire used was explicitly developed for patients with physical illnesses, but only allows global statements regarding perceived social supports. Due to the global approach of measuring perceived social support, it is not possible to identify the sources of social support. Due et al. [38] showed significant age differences in the structure and function of social relations. Thus, it can be assumed that social networks are different between and within the two age groups. Information about which people the patients thought of when answering the questionnaire was not collected. Based on the analyzed data, we are unable to make definitive statements about differences between AYA patients and older patients as compared to middle-aged haematological cancer patients (41 to 69 years). Due to the cross-sectional design of the study, we are also unable to report changes of perceived social support over time.

### Future research

Future studies should examine AYA and older adult patients’ social networks and their impact on patients’ social support chosing a source-specific approach. Whether positive and negative social support is provided by the same people within a patient’s social network should also be clarified. An initial indication for this phenomenon was observed in Breuer et al.’s [43] qualitative study, whereby AYA patients described receiving both positive and negative social support from their partner, family and friends. Furthermore, future research should give more attention to negative social support and examine whether positive social support acts as a buffer against negative social support. Although we tried to control for these differences in the regression models, it remains unclear whether age and/or being in a partnership are relevant social support factors. This should be investigated in future studies.

Moreover, longitudinal studies should be conducted to examine changes in positive and negative social support. We also need to learn more about similarities and differences in cancer patients’ social networks at different points in the human lifespan and the course of cancer disease.

## Conclusion

Young and older haematological cancer patients reported high levels of positive social support. The differences in levels of perceived negative social support between the two patient groups and its impact on health-related quality of life emphasise the need to examine not only positive but also negative aspects of social support. Special attention should be given to negative social support when providing psychosocial care to young patients in order to help improve their health-related quality of life.

## Data Availability

The dataset which the study is based on is publicly not available due to required data protection but is available upon reasonable request with a signature of a data privacy form. To request the data, the reader may contact the first author. The study received research ethics committee approval (file number: 372–13-16122013 and 071–14-10032014)) from ethic board of the medical faculty of the University of Leipzig. Informed consent was obtained from all participants included in the study.

## Abbreviations

AYA: young adult patients
EORTC QLQ-C30: European Organisation for the Research and Treatment of Cancer Quality of Life Questionnaire-Core 30
HRQoL: Health-related quality of life
ISSS-8: Illness-specific Social Support Scale short version-8
